# A Rare Presentation of Donkey Bites Involving the Cheek and Ear: A Case Report and Literature Review

**DOI:** 10.7759/cureus.37446

**Published:** 2023-04-11

**Authors:** Nancy Zeaiter, Deoda Maassarani, Charbel B Aoun, George Ghanime, Ziad Sleiman

**Affiliations:** 1 Plastic and Reconstructive Surgery, Lebanese University, Beirut, LBN; 2 Plastic and Reconstructive Surgery, Lebanese Hospital Geitawi UMC, Beirut, LBN

**Keywords:** animal bite, animal bite management, case report, literature review, ear, cheek, donkey bite

## Abstract

Although animal bites account for a fair number of emergency department visits, donkey bites account for a very limited proportion. A 12-year-old boy presented to our department with a severe donkey bite involving his face. The injury included his left cheek with a laceration of the left ear cartilage. The examination revealed no serious morbidity (no vascular or nerve involvement). The patient received prophylactic antibiotics and anti-rabies/anti-tetanus vaccination. The wound was cleaned thoroughly with copious irrigation. Afterward, the patient underwent surgery to correct the defect in the cheek using a rotational advancement cervicofacial flap, while the penetrated ear cartilage was repaired and the skin margins were approximated and sutured. During the follow-up period, no complications were observed and the functional and cosmetic outcomes were satisfactory. Donkey bites are rarely encountered and they can result in different presentations and morbidities/outcomes. It is suggested that the timing from the bite injury to presentation, the stage/extent of the bite, the use of anti-tetanus and anti-rabies vaccines, and the prophylactic use of antibiotics may play a role in determining the outcomes and/or complications of donkey bites.

## Introduction

Mammalian bites account for 1% of all visits to the emergency room, making bite wounds rather common. Dog, cat, and human bites are increasingly common and are a significant cause of morbidity and mortality [1]. Based on a previous report, around 50% of Americans will experience an animal or human bite at least once during their life span, and 45% of children had been bitten at some point [2]. An animal bite could have an impact on any part of the human body, including the upper extremity [3], lower extremity [4], thorax [5], female breast [6], and genitalia [7]. Frequently, the head and neck area is involved [2]. Animal bites are a significant issue in health care and frequently result in trauma to the hands and faces of young children.

However, donkey bite injuries are fairly uncommon, despite having the potential to cause serious injuries and complications [8]. The associated morbidity and mortality are widely variable depending on the location and extent of the bite; however, bite-associated outcomes are frequently worse in horse bites than in donkey bites. Particularly, maxillo-facial trauma caused by donkey bites is uncommon with more than 75% of the facial bites affecting the lips, nose, or cheeks [9]. It can result in serious, and sometimes fatal, facial injuries with profound functional and cosmetic consequences [9].

The management of animal bites includes both local wounds and systemic considerations [1]. It can be divided into general and specified approaches [1]. The general approach includes a thorough examination and proper cleaning of the wound. Meanwhile, the specified approach depends mainly on the site and extent of the bite as well as associated injuries involving the adjacent nerves or arteries. Antibiotic consideration is an essential step in the management process to prevent wound infection, which is a serious complication with potentially negative functional and/or cosmetic outcomes.

Even though donkey bites may resemble other mammalian bites in terms of clinical presentation, there are specific features that distinguish these bites. Donkey bites may be circular or oblong in shape, with a diameter ranging from a few millimeters to several centimeters. They may also have a puncture wound at the center. Furthermore, they can be quite powerful and can result in deep tissue damage. Unlike other animal bites, such as dog bites, which tend to be quick and sudden, donkey bites are often sustained over a longer period, which can result in more extensive injuries [10].

Here, we present a case of a donkey bite-associated cheek and ear laceration with special emphasis on its presentation, management, and postoperative outcomes.

## Case presentation

**Figure 1:**
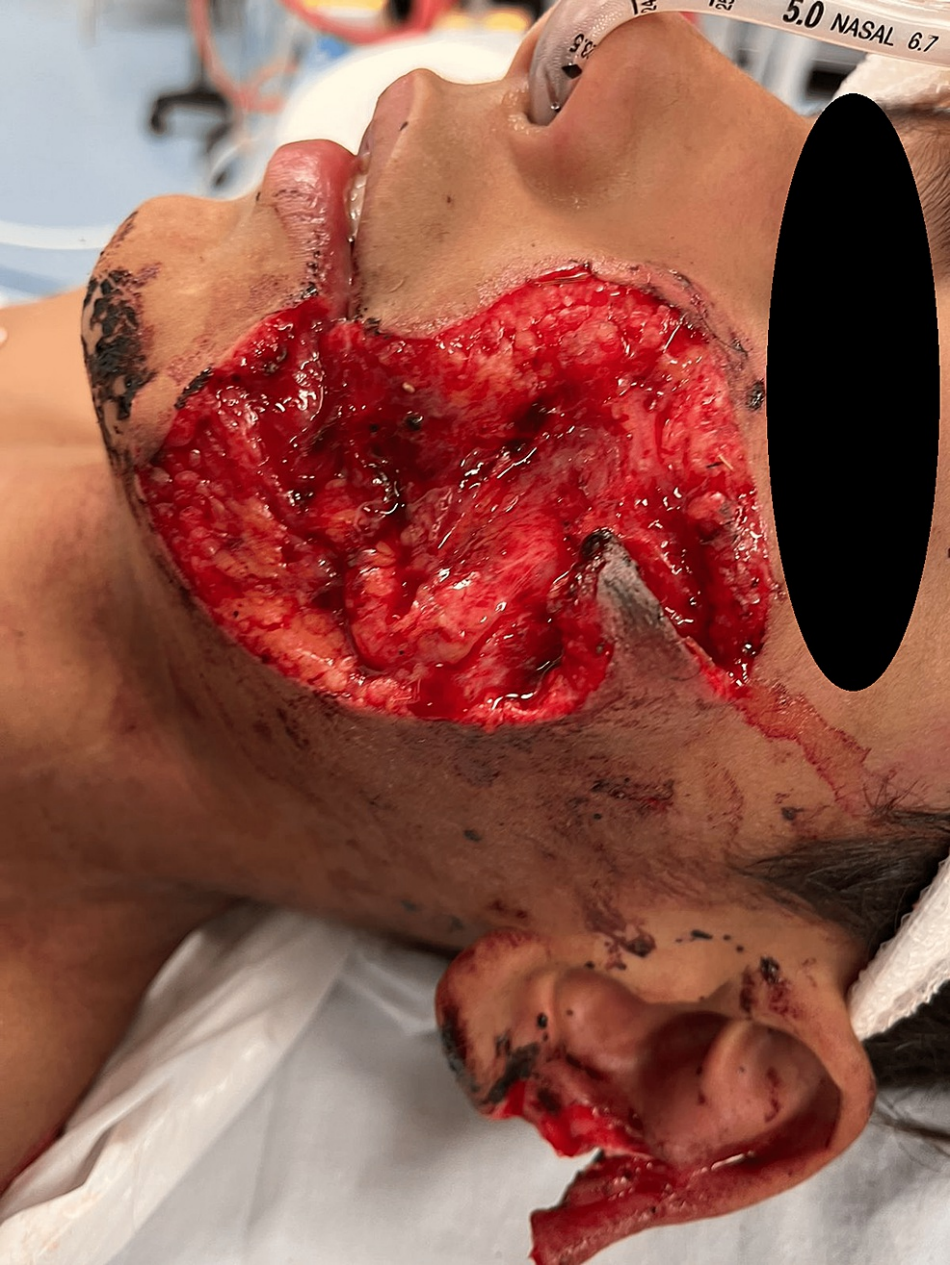
Post-injury wound (one hour after the bite), showing bite marks on the left cheek and ear

A 12-year-old boy was admitted to the Emergency Department at Lebanese Hospital Geitawi, Beirut, Lebanon. One hour after the incident, the patient presented with a severe donkey bite involving the face, resulting in left cheek and ear lacerations (Figure 1). The patient in this case provoked the donkey by trying to touch it without the owner's permission. The donkey, as a result, became agitated and bit the patient. It is noteworthy that the patient consented to the use of his charts and images for the purposes of this report.

The patient's medical history revealed no significant findings, and he was completely healthy prior to the incident. A detailed physical examination was carried out, showing the following findings: no intra-oral communication with the cheek laceration, inability to elevate the left angle of the mouth in a superolateral direction, and inability to smile. Meanwhile, the patient was able to show his teeth, blow out his cheek, whistle, close his eyes completely against resistance, and wrinkle his forehead. Upon examining the patient’s left ear, we found lacerations of the external part of the ear (on the helix and anti-helix) while the cartilage was penetrated. However, his injury did not affect his hearing capacity; both ears had the same capacity.

**Figure 2:**
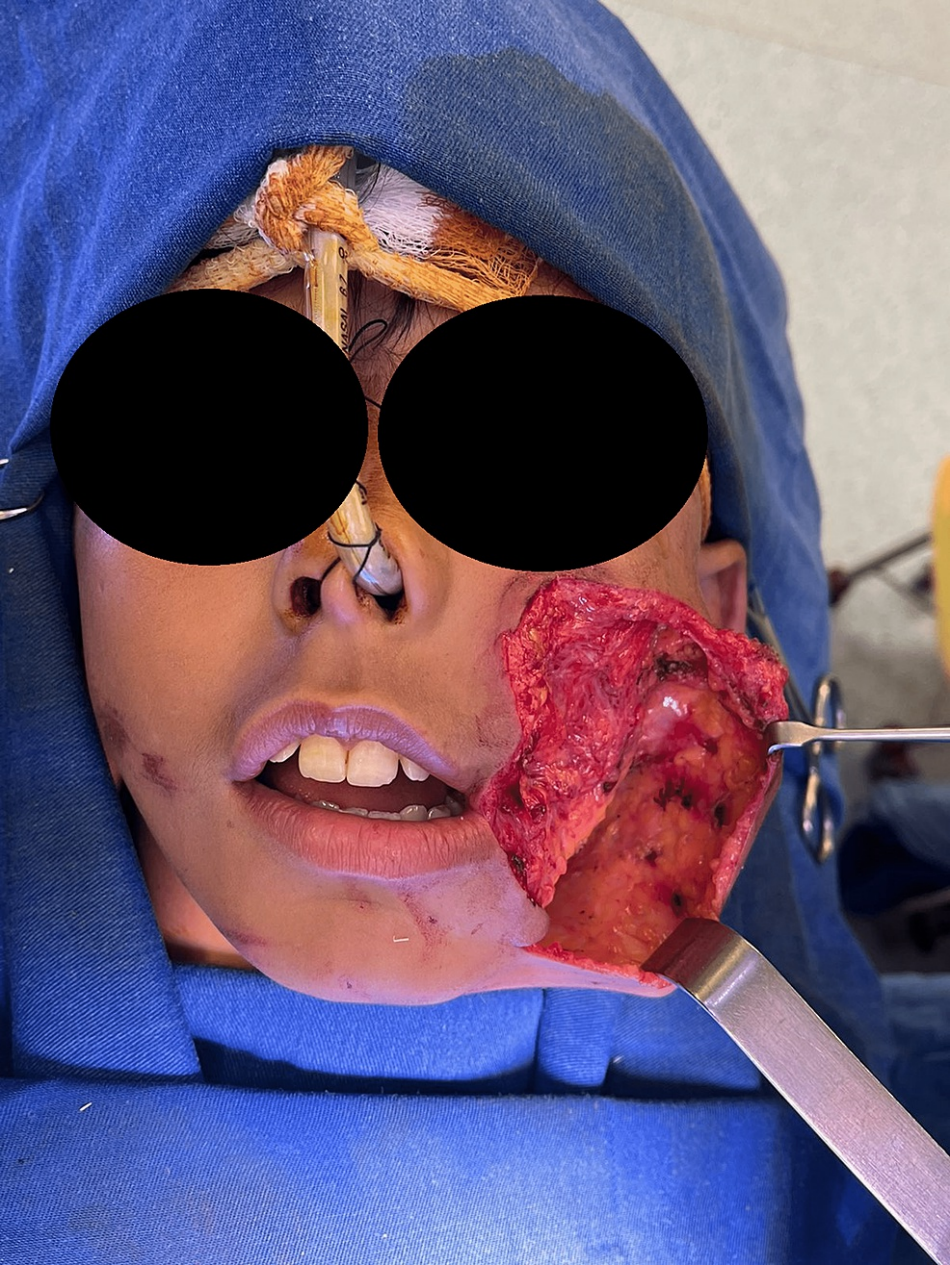
Exploration of the wound and dissection of the flap

**Figure 3:**
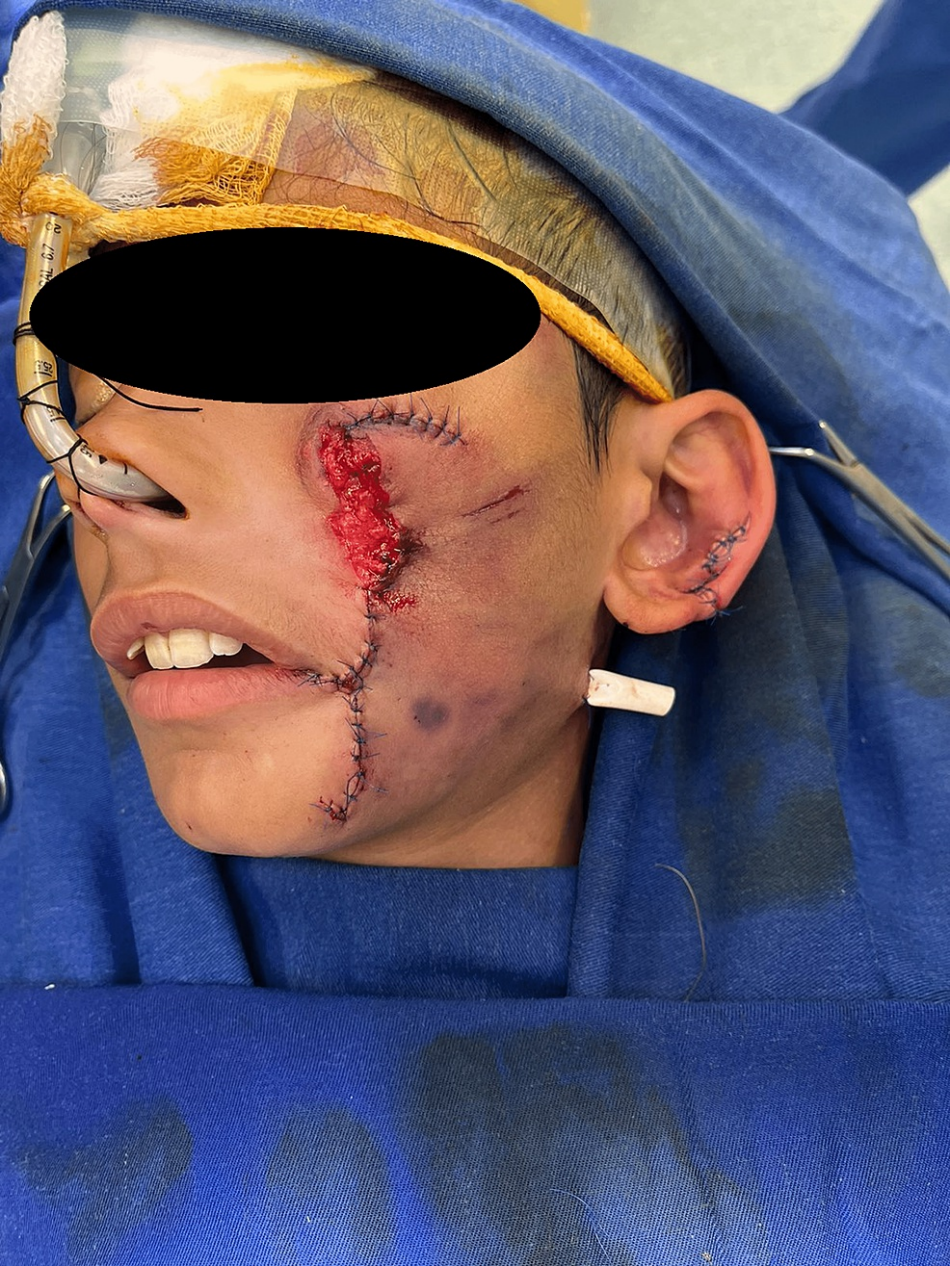
Immediate postoperative results

The injury was then managed immediately and appropriately. Copious irrigation and proper cleansing were done using normal saline. Afterward, the patient received antibiotics (ceftriaxone 1g once daily and clindamycin 600mg twice daily) as well as tetanus and rabies prophylaxis. The patient was then taken to the operating room, where proper cleaning of the wound was carried out under general anesthesia. Exploration of the defect in the left cheek was done (Figure 2), revealing a partial loss of the zygomaticus major muscle (mainly at its insertion site); however, the facial nerve was completely intact at this point. Stensen’s duct was intubated and found intact. In order to cover the site of the defect, a rotational advancement cervicofacial cheek flap (Mustarde flap) was done. Similarly, the penetration of the ear cartilage was repaired and then the skin margins were sutured (Figure 3). 

**Figure 4:**
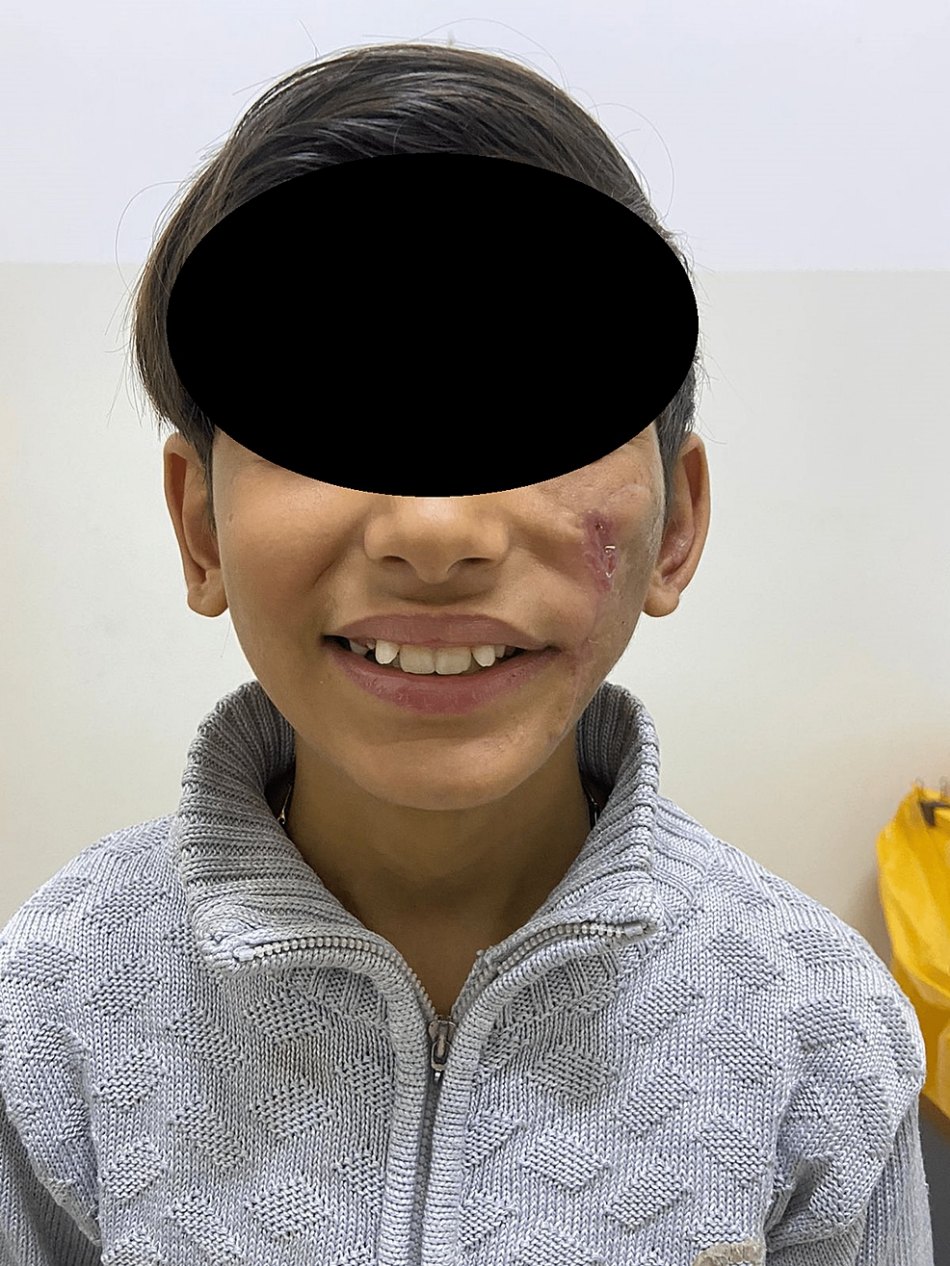
Result after four months of follow-up, frontal view

**Figure 5:**
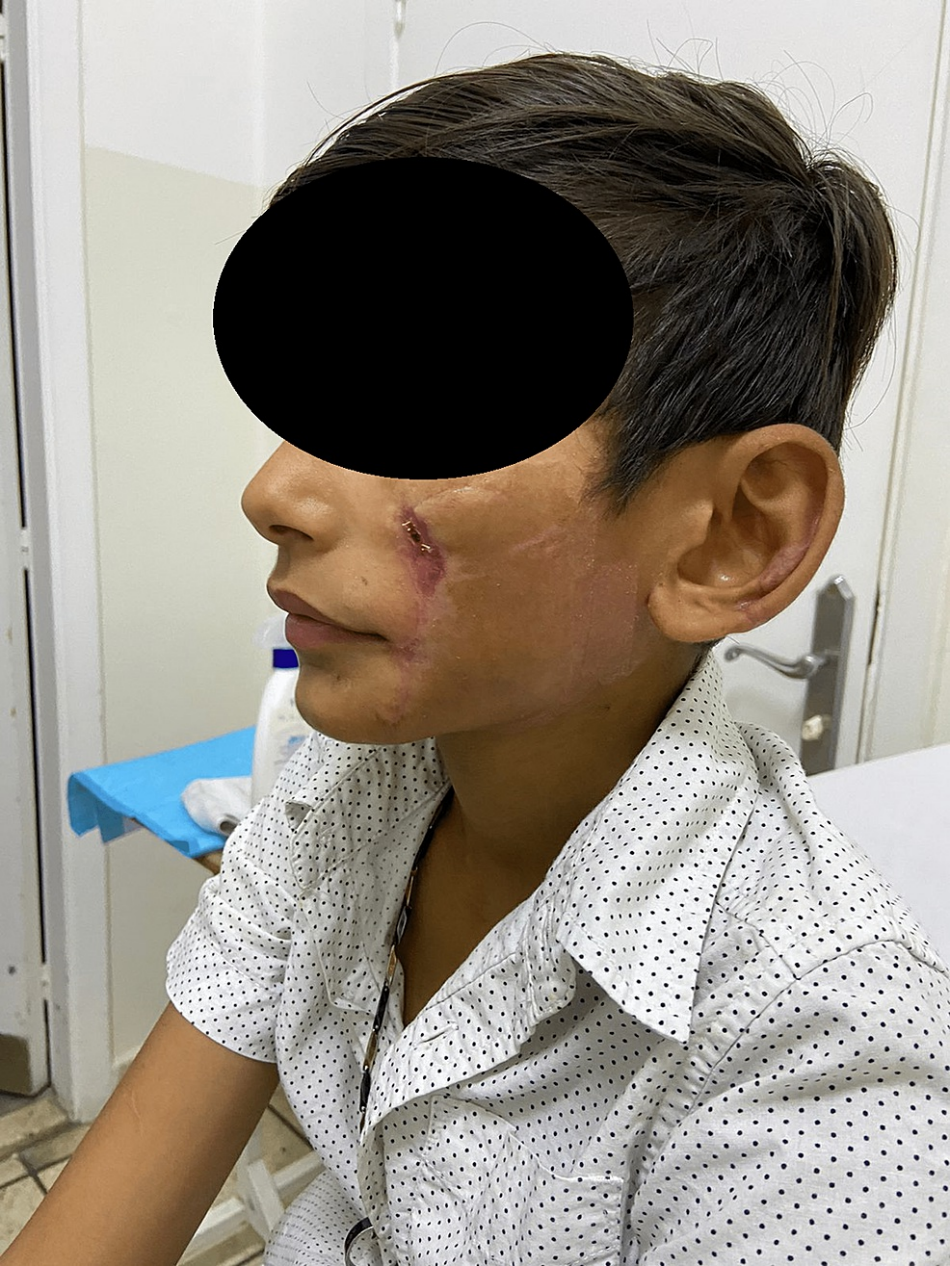
Result after four months of follow-up, profile view

Following surgery, the patient remained on the same regimen of antibiotics for five days. During the postoperative period (after four weeks), all wounds healed satisfactorily without any complications or flap necrosis. Figures 4-5 show satisfactory frontal and profile pictures, respectively, at the four-month follow-up.

## Discussion

To date, there are no accurate statistics regarding the incidence or rate of animal bites, particularly donkey bites, around the globe. However, a recent chart-review study in Turkey reported an incidence of horse and donkey bites of 7.8 per 100,000 individuals. Upon reviewing 445,698 records over three years, only 36 cases of donkey and horse bites combined were reported; of those only 26 cases were specific to donkey bites [10].

Despite being a rare event, the presentation of donkey bite can vary substantially, depending on numerous factors, including age and the site and extent of the bite (Table 1). Upon reviewing the literature, we found that cases of donkey bites were more commonly reported in males (17 out of 20 cases) and in places where donkeys were raised as domestic animals (nine out of 20 cases), and this, in turn, can potentially result in an increased likelihood of bites secondary to the prolonged exposure to animals. The age of patients can play an important role in the occurrence of these incidents. Cases of animal bites have only been reported in the extremes of age (very young or very old). Among the 20 cases reported in the literature, the age ranged from five [11] to 76 [12]. This occurrence of donkey bites in these cases is, hypothetically, justifiable given their inability to defend themselves from any attacks or for doing something that can provoke these animals [10].

**Table 1:** The characteristics of donkey bites as reported in the literature

Author, year of publication	Case	Age	Gender	Animal Type	Medical History	Injury Site	Time to presentation	Examination Findings
Österlund et al., 1996 [8]	Case one	20	Female	Domestic	Healthy	Left thumb	-	Pain, teeth marks, abraded area on the volar side (2 x 3 cm), no fractures or joint injuries
Shipkov et al., 2004 [9]	Case one	67	Male	-	-	Nose - Lip	Six hours	Poor condition (indicative of a hypovolemic shock), total nasal amputation, defect in upper lip
	Case two	70	Male	-	-	Nose - Forehead	-	Laceration in the forehead, amputated nose
Droussi et al., 2014 [11]	Case one	Three	Female	Domestic	Healthy	Scalp	11 hours	No neurological deficit, no fractures, open injury of scalp including the periosteum (12 cm in length)
	Case two	Nine	Male	Domestic	Healthy	Scalp	Four hours	No neurological deficit, no fractures, open injury of scalp including the periosteum (10 cm in length)
	Case three	Five	Male	Domestic	Healthy	Scalp involving the skull	Five hours	Deep, open wound (12 cm of scalp tissue and 4 cm of the parietal bone with meningeal involvement), subarachnoid hemorrhage
Eshraghi et al., 2017 [12]	Case one	76	Male	-	-	Face (eye)	-	Right eye: lower eyelid completely avulsed/eye completely lost. Left eye: superior and inferior lid eyelid margins were lacerated/full-thickness penetrating laceration
Bloch et al., 1976 [13]	Case one	2.5	Male	Domestic		Face - Ear - Feet	-	Conscious, no hemorrhage, lacerated wounds (face), tear (left ear)
Fogel et al., 2018 [14]	Case one	65	Male	-	-	Face - scalp	-	Dead on examination, contusions and lacerations of face/scalp, fracture of left maxilla and right radius and ulna, bite marks on left thigh, right buttock, right upper arm, and left cheek
d'Aloja et al., 2011 [15]	Case one	65	Male			Neck	-	Dead on examination, very deep wound in the anterior part of the neck, deep contusion in the right shoulder
Köse et al., 2010 [16]	Case one	-	-	-	-	Neck	-	-
	Case two	-	-	-	-	Neck	-	-
	Case three	-	-	-	-	Neck	-	-
	Case four	-	-	-	-	Extremity	-	-
	Case five	-	-	-	-	Neck	-	-
Mosbahi et al., 2020 [17]	Case one	Two	Female	Domestic	Healthy	Neck - Shoulder	-	Dead on examination, bruises and deep injuries "bite marks" in the right arm, purplish arc-shaped bruise in right earlobe and tragus, bite marks on neck and upper shoulder
Tiemdjo et al., 2009 [18]	Case one	13	Male	Domestic	Healthy	Left leg	Two days	Tibiofibular fracture (no vascular or nerve damage), no systemic symptoms (i.e., fever)
	Case two	11	Male	Domestic	Healthy	Left leg	Two hours	Conscious, good general condition, apyretic, tibiofibular fracture (no vascular or nerve damage)
De Luca et al., 2017 [19]	Case one	Seven	Male	-	-	Penis	-	Partial penile amputation, intact scrotum and testes
Ouattara et al., 2020 [20]	Case one	15	Male	Domestic	-	Penis	-	Penile glans amputation and hemorrhage

Reported donkey bites can involve various body parts, including the face (five cases) [9,12-14], the neck (six cases) [15-17], extremities (six cases) [13,16,18,19], the ear (one case) [13], the scalp (three cases) [11] or the penis (two cases) [19,20]. Our case came to the emergency department with rather a rare presentation involving the cheek and ear, which are rarely reported in the literature. The presentation and associated morbidity are dependent on the classification of the injury. The most commonly used classification system is Lackmann’s classification of bite injuries (Table 2) [11]. Our case presented with stage three, a deep wound involving the muscles with the presence of a defect that was observed upon exploration. The stage of donkey bites in the literature have varied widely, and there are cases where bone involvement/fracture [18] or organ amputation [19,20] have been reported. Furthermore, three cases have been found dead on examination following donkey bites [14,15,17]. The outcomes and associated morbidity of the donkey bite are linked with the stage of the bite injury.

**Table 2:** Lackmann’s classification system of bite injuries Source: Droussi et al., 2014 [11]

Staging	Extent of Injury
Stage I	Superficial wound without muscle damage
Stage II	Deep wound with muscle damage
Stage III	Deep wound with muscle damage and defect
Stage IVa	Stage III with vascular or nervous damage
Stage IVb	Stage III involving bone or amputation

The management of donkey bites is dependent upon the site and extent of the bite. However, generally, the management of such bites is done in two steps: general wound cleaning and irrigation and then specific management (surgery if needed) to correct any associated defects. The detailed management protocol of such cases in the literature is summarized in Table 3. The aim of treatment is to reach optimum functional as well as cosmetic outcomes. The timing from bite injury to presentation, the use of anti-tetanus and anti-rabies vaccines, and the use of prophylactic antibiotics might aid or quicken the healing process (Table 3). Post-management complications are not rare, and they may range from simple minor complications (such as minor scars) [16] to moderate complications (extensive scarring and/or infection) [9] to serious complications (such as death probably due to fat embolization) [13]. In our case, the injury was healed satisfactorily without any complications. This might be related to the proper management (surgical exploration, antibiotic use, and rabies and tetanus vaccination) given to the patient in a timely manner upon presenting within one hour of the bite injury incident. It has been observed in the literature that donkey bites involving the upper extremity or the digits ended in a purulent infection and tissue necrosis [8,16]. In these cases, no prophylactic antibiotic was administered, and patients were not vaccinated against rabies and tetanus upon presentation [8,16]. In terms of cosmetic outcomes, the timing from the bite injury to presentation has been hypothesized to play a role. In the two cases reported by Tiemdjo, et al., donkey bites involved their legs [18]. In one case, the patient retained his functional capacity with normal movement and the wound healed completely. Meanwhile, in the second case, the healing process of the bone took longer, the patient regained his normal range of motion within 270 days, and the wound healing was of poor quality. This could be because of the timing from the bite to presentation and appropriate intervention; in the first case the patient presented within two hours, while in the second case presented within two days after the bite.

**Table 3:** Management protocol and postoperative functional and cosmetic outcomes of donkey bites in the literature FTSG: full-thickness skin graft

Author, Year of Publication	Case	Vaccination	Injury Management					Outcomes			Management of complication
			General	Antibiotic		Specific		Immediate	Follow-up observations	Timing	
Österlund et al., 1996 [8]	Case one	Tetanus	-	-				No healing	Purulent infection	Five days	Oral cefadroxil 500 mg twice daily (15 days)
Shipkov et al., 2004 [9]	Case one	-	-	Ceftriaxone (2g/24h), metronidazole (1g/24h) for five days		Lip: primary suturing. Nose: covered by an oblique forehead flap. Cutaneous defect: covered by a supraclavicular FTSG		Functional: satisfactory. Cosmetic: uneventful wound healing	No infection	-	-
	Case two	-	-	-		Secondary repair of the amputated nose		Functional: satisfactory. Cosmetic: uneventful wound healing	Scarring	Six months	Three-stage nasal reconstruction
Droussi et al., 2014 [11]	Case one	Tetanus	-	Amoxicillin-clavulanic acid (50mg/kg, three times/day for one week)		Debridement and coverage with occipital scalp flap			Appropriate wound healing	30 days	-
	Case two	Tetanus	Wound care and dressing changes	Amoxicillin-clavulanic acid (50mg/kg, three times/day for one week)		A split-thickness skin grafting (one month)		-	Appropriate wound healing	30 days	-
	Case three	Tetanus	-	Amoxicillin-clavulanic acid (50mg/kg, three times/day for 15 days)		Debridement with the aponeurosis of the fascia lata, coverage by a rotation flap		-	-	-	-
Eshraghi et al., 2017 [12]	Case one	Tetanus - Rabies	Copious irrigation	Yes		Left eye: globe repair. Right eye: evisceration		Bilateral vision loss	-	-	-
Bloch et al., 1976 [13]	Case one	Tetanus	Cleaning - Disinfection			Suturing (facial wounds), plastic repair (ear), plaster of Paris (for the fracture)		Death	Fat embolism	Two days	-
Fogel et al., 2018 [14]	Case one	-	-	-		-		Death	-	-	-
d'Aloja et al., 2011 [15]	Case one	-	-	-		-		Death	-		-
Köse et al., 2010 [16]	Case one	-	Wound cleansing (saline)	None		Debridement and suturing (primary)		-	Minor scar complications	-	-
	Case two	-	Wound cleansing (saline)	None		Debridement and suturing (primary)		-	Minor scar complications	-	-
	Case three	-	Wound cleansing (saline)	None		Debridement and suturing (primary)		-	Minor scar complications	-	-
	Case four	-	Wound cleansing (saline)	None		Debridement and suturing (primary)		Tissue necrosis and infection	-	-	-
	Case five	-	Wound cleansing (saline)	None		Debridement and suturing (primary)		-	-	-	-
Mosbahi et al., 2020 [17]	Case one	-	-	-		-		Death	-	-	-
Tiemdjo et al., 2009 [18]	Case one	Rabies	Emergency wound care, wound dressing	Parenteral amoxicillin (1g twice daily - six days)		Surgical exploration, wound cleansing and debridement, circular full-leg cast with a window at the wound	-		30 days: bone consolidation - tibiofibular synostosis. Eight months: Functional: walking, painless, no limping, and normal range of motion. Cosmetic: wound healing was of poor quality	30 days	-
	Case two	Tetanus - Rabies		Parenteral amoxicillin		Surgical exploration, wound cleansing			Functional: no pain or limping and normal range of motion/bone healed. Cosmetic: wound healed	28-70 days	-
De Luca et al., 2017 [19]	Case one	-	-	-		Total phallic reconstruction (at age 23)		-	-	-	-
Ouattara et al., 2020 [20]	Case one	Tetanus	-	-		Hemostatic suturing, stump regularization, and penile glans reconstruction surgery		Functional: satisfactory - good quality of micturition. Cosmetic: satisfactory	No complications	Six months	-

## Conclusions

Donkey bites are rarely encountered and they can result in different presentations and morbidities/outcomes. It is suggested that the timing from the bite injury to presentation, the stage/extent of the bite, the use of anti-tetanus and anti-rabies vaccines, and the prophylactic use of antibiotics may play a role in determining the outcomes and/or complications of donkey bites. Therefore, prompt and appropriate medical attention along with proper wound management are essential in minimizing the potential risks associated with donkey bites.
